# Evaluating UK Pharmacy Workers’ Knowledge, Attitudes and Behaviour towards Antimicrobial Stewardship and Assessing the Impact of Training in Community Pharmacy

**DOI:** 10.3390/pharmacy10040098

**Published:** 2022-08-16

**Authors:** Donna Seaton, Diane Ashiru-Oredope, Jordan Charlesworth, Isla Gemmell, Roger Harrison

**Affiliations:** 1 Division of Population Health, Health Services Research & Primary Care, The University of Manchester, Manchester M13 9PL, UK; 2 HCAI, Fungal, AMR, AMU & Sepsis Division, UK Health Security Agency, London SW1P 3JR, UK

**Keywords:** antibiotic resistance, service evaluation, antibiotic stewardship, antibiotic guardian, public health campaign, behaviour, COVID-19

## Abstract

The Antibiotic Guardian (AG) campaign, developed in 2014 is an online ‘pledge’ approach to engage health workers and the public about antimicrobial resistance. It is underpinned by models of science communication and behaviour change. Since its launch until the end of 2021, more than 140,000 individuals pledged. A service evaluation was conducted to determine the impact of the campaign upon UK pharmacy workers, in response to national training introduced in 2020. Pledged pharmacy workers were sent an online questionnaire collating demographics, self-reported behaviour and opportunity to support prudent antibiotic use. It also investigated respondents’ daily practice and antimicrobial stewardship (AMS) efforts, and motivations for pledging. Capability was measured with a set of knowledge questions. Awareness of changes to the Community Pharmacy Quality Scheme in England to include incentivized training on antimicrobial resistance (AMR) was explored. Of the 5344 pharmacy workers invited to participate, 783 (14.6%) responded to the survey. There was a statistically significant difference between job roles and capability score. Pharmacists, including Academic and Hospital Pharmacists and Pharmacy Technicians reported higher confidence and capability scores than Dispensers and Pharmacy Assistants (F = 13.776, *p* = 0.0002). Respondents reported strong knowledge on antimicrobial resistance and high confidence in fulfilling their AG stewardship pledge within daily practices (92.7% of all respondents answered all capability questions, as measured by knowledge, correctly). Two thirds of respondents (61.6% (423/693)) agreed or strongly agreed that they had access to and were able to utilise local antibiotic prescribing guidance and a similar proportion of responding community pharmacists (60%) were aware of the content of their workplace AMS plans. No statistically significant relationships were found between motivations for pledging and subsequent behaviour; pledging due to mandatory requirements of work-place training was the most common answer in both 2019 (42%) and 2020 (54%) cohorts. This evaluation supports the value of the AG pledge-based approach to engage and educate pharmacy workers. Reflections show its impact on increasing evidence-based stewardship for pharmacy workers and their response to mandatory training requirement by employers highlights the effectiveness of the AG campaign to promote AMS within pharmacy teams.

## 1. Introduction

Until the early 20th century, infection was the leading cause of death in humans; based on historical available data, what are now considered easily treatable bacterial infections or symptoms of infections, such as pneumonia or diarrhoea, were once leading causes of mortality and morbidity worldwide [1]. The development of antibiotic therapy was a breakthrough in the fight against bacterial infection across the globe [2]. However, since the 1970s no new class of antibiotics have been brought to market [3]. Recent antibiotic development has involved modifications or isomers of existing empirical formulas, or the development of combination antibiotic therapy. Whilst there exists an abundance of research into the development of more novel infection treatment, human healthcare continues to rely dominantly on antibiotics. Consequently, increasing rates of antibiotic use are a major threat to health, with resistance even developing to combination and third-line antibiotics.

Bacterial pathogens are adaptable to their environment and possess the ability to create physiological and biochemical changes within themselves [4]. This has led to the development of antimicrobial resistance (AMR). It is estimated that AMR is responsible for 700,000 deaths worldwide each year [5]. In the UK, pharmacies, being distributors and dispensers of antibiotics, are ideally placed to ensure correct prescribing, provide professional clinical advice, and give understandable information and direction to patients, including answers to their concerns [6].

International and national action plans highlight the need for continued professional and public engagement as one approach to reduce the unnecessary use of antibiotics, and of those used, to ensure they are used and disposed of correctly. In repose to this, the Antibiotic Guardian (AG) was established as an international campaign developed in 2014 by Public Health England (PHE) (now UK Health Security Agency) in collaboration with the Department of Health’s Expert Advisory Committee on Antimicrobial Resistance and Healthcare Associated Infections (ARHAI) and the Department for Environment Food and Rural Affairs (DEFRA) [7]. The AG campaign seeks to engage and promote action in response to AMR, of which a key component is the clinically appropriate dispensing and correct usage of antimicrobials by patients [8]. A process evaluation in 2016 reported ongoing brand visibility of the campaign internationally. Between 2014 and 2016, there were 47,158 unique visitors to the website across 156 different countries. Between August 2014 and January 2015, there were 12,086 ‘tweets’ on the social media platform Twitter that used the #AntibioticGuardian hashtag [9]. From its initiation in 2014 to the end of 2021, there have been 144,446 pledges on the main AG webpage. Pledges were predominantly made by those identifying as a health/social care professionals or leaders (94,122 pledges); of these, 68,192 were made by those belonging to Pharmacy Teams, including from primary and secondary care and community pharmacy. Between 2014 and the end of 2021, 36,894 of those that pledged stated finding out about AG through community pharmacy (Communication from Antibiotic Guardian Team).

The Pharmaceutical Services Negotiating Committee, who support and advocate for all NHS pharmacies in England, reports that there are currently more than 11,200 community pharmacies operating within the UK. Furthermore, within England, 90% of the population live within a 20 min walking distance of a community pharmacy. Within areas of high deprivation, this increases to almost 100% [10]. These figures reflect the potential value of antimicrobial stewardship (AMS) from community pharmacies, with accessibility and identity of clinical intervention a key component of service provision. Two years after the AG campaign was established, NHS England and NHS Improvement developed the Quality Payment Scheme in 2016 for community pharmacies [11]. This was later rebranded the Pharmacy Quality Scheme (PQS). The scheme followed the principles of the Community Pharmacy Contractual Framework to incentivise the delivery of quality clinical effectiveness, patient safety and patient experience. In response to the NHS Long Term Plan, and to bridge the gap between pharmacy workers’ knowledge on AMR and their subsequent stewardship, as well as to tackle infection control during the COVID-19 pandemic, NHSE launched the Pharmacy Quality Scheme: Part 2 in 2020 [12]. This scheme included a domain exclusively for Infection Prevention and Control, with a specific AMS section. As part of the domain, there was a component for pharmacy staff to become AG (amongst other related criteria—Box 1) [13]. The aim of this PQS criterion was to ‘further support vital work to limit AMR through effective AMS activities through community pharmacy, influencing responsible antibiotic prescribing and patients’ personal attitudes and social norms around use of antimicrobials’. 

This project sought to provide an evaluation and analysis of the AG campaign to understand its impact amongst pharmacy workers. Furthermore, it sought to evaluate the motivations for pledging of community pharmacy workers and the impact of the PQS. This evaluation was underpinned by an evidence-based model of behaviour change [14], seeking to determine the relationship between individuals’ capability, opportunity and behaviour with regards to antimicrobial stewardship and reasons for making an online pledge. The project explored the impact of motivation to pledge to be an AG on subsequent capability (as measured by knowledge) and behaviour. It also assessed the changes prior to and following the introduction of the Pharmacy Quality Scheme addition of the Infection Prevention and Control domain.

Box 1 **PQS requirement [11].**

On the day of the declaration all pharmacy staff must have satisfactorily completed the PHE Antimicrobial Stewardship for Community Pharmacy e-learning on e-learning for Healthcare and e-assessment.

In addition, contractors must have available, at premises level, the antimicrobial stewardship action plan for the whole community pharmacy including how they will promote Antimicrobial Stewardship (AMS). The action plan must demonstrably include details of how all staff involved in the provision of self-care advice will incorporate the principles of AMS in to self-care advice, including reinforcing the messages around appropriate use of antibiotics, uptake of vaccinations, including the influenza vaccination, becoming antibiotic guardians and having an awareness of the local antibiotic formu-lary.

There must be demonstrable evidence, at the pharmacy, that the actions have been implemented by the day of the declaration.

## 2. Methods

This was a cross-sectional study, using a self-completion questionnaire to collect data from individual pharmacy workers across the UK that had pledged to become AGs. The questions included those validated in previous evaluations on aspects of the AG and were informed by a wide literature review in relation to health professional communication and engagement. A number of questions were used to collect information in relation to behaviour change [14]. Data was also collected with respect to professional background and demographics. It was piloted with 20 pharmacy workers, made up of pharmacists, pharmacy technicians and health care assistants self-recruited by one of the researchers (DS) prior to deployment. In addition, Cronbach’s Alpha scores were calculated to test convergent validity and the internal consistency of the questions. Each question set followed a different answer system or strategy. A mix of Likert scales, tick boxes and a sliding scale were utilised. The questionnaire was reviewed by members of the World Antibiotic Awareness Week and European Antibiotic Awareness Day Planning Group for Public Health England. The final questionnaire consisted of 13 questions and was built using Google Forms (Supplementary Material).

The question subset blocks consisted of the following:

Demographic Questions: At beginning of the survey to collate respondents’ general non-identifiable background information (4 questions in the subset);

Capability: Knowledge on human antibiotic health facts (14 questions in the subset);

Opportunity and Attitude: Self-report questions around knowledge of local guidelines, access to health information and current practice (7 questions in the subset);

Motivation: Individual reasons for making a pledge to be an Antibiotic Guardian (5 questions in the subset);

Behaviour: Advice given to patients/prescribers in the last week, local formulary use, health promotional materials and AMS (6 questions in the subset);

Awareness: Knowledge of WAAW, changes to Pharmacy Quality Scheme, workplace health promotion and access to resources (4 questions in the subset).

The full set of questions in the survey is available in Supplementary Material S1.

People making their online pledge for the AG campaign had an opportunity to enter their email address to receive a confirmation of their pledge and certificate. They could also provide consent for future contact about their pledge (Supplementary Figure S2). This email was used to sample then send invitations, including informed consent and a link to the online questionnaire (for those who had given consent for future contact about their pledges).

The questionnaire was sent out to a cross section of pledgers from World Antibiotic Awareness Week (WAAW) in November 2019 and November 2020. A reminder was sent one week later. The survey was open from 18 June 2021 to 16 July 2021. Based on the University of Manchester’s ethics decision tool (Supplementary Figure S1), ethical approval was not required as this project was a service evaluation and participants had previously agreed to be contacted about their pledges for future evaluation or training opportunities. In addition, no personal information was received by any researcher outside of the AG team. Approval for consent following receipt of the invitation letter and link to the survey was then sought (Supplementary Figure S3). Upon receipt of the survey, the respondents’ emails were deleted from the dataset by the AG team to maintain anonymity prior to sending to DS for analysis.

Basic descriptive statistics were completed and relationships between behaviour change, policy implementation and pharmacy workers’ attitudes and behaviour with response to AMS were assessed using independent *t*-tests and ANOVA or Kruskal–Wallis if the data did not meet the assumption of ANOVA. Inferential statistical analysis used IBM SPSS Statistics: Version 25 [15] and statistical significance was indicated with *p* < 0.05.

## 3. Results

### 3.1. Demographics

During the months of November 2019 and November 2020, 5338 pharmacy workers had made a pledge and given permission for further contact. They were sent an invitation email to participate in the study and a link to the online questionnaire. Across the four-week study period, (18 June 2021 to 16 July 2021) 783 (14.6%) replies were received. One respondent was no longer working in pharmacy and 15 declined to complete the survey leaving 767 eligible participants. Of the eligible participants 429 (55.9%) had pledged to be an AG in 2019 and 338 (44.1%) in 2020. In total, 24.4% (187/767) of respondents were between the ages of 46 and 55, and almost all respondents were between 18 and 65 years old. Any of the respondents in the 18 or less age range responded as being 18 years old or under. The majority were female (80.2% (615/767) and 35.9% (275/767) had been working or been registered as pharmacists or pharmacy technicians for between 3 and 10 years. As shown in Table 1, respondents were predominantly Community or Primary Care Pharmacists (42.9%, 329/767), Pharmacy Technicians (17.2%) or Dispensers (17.7%).

### 3.2. Capability

Respondents were asked to answer eight questions to determine their knowledge, responding as true or false. Each question was allocated a score of 0 or 1, and a total summary score (maximum 8/8) calculated for all eight questions. For all 767 respondents the mean score was 7.1. The highest mean score was for the job role category of ‘Pharmacist’ (mean 7.4, SD = 0.9, range = 4) with Dispensers scoring the lowest (mean 6.46, SD = 1.6, range = 6). All responding as Academic Pharmacists (*n* = 4) answered all eight questions correctly. 

Respondents who had pledged in 2019 achieved a higher mean knowledge score (mean = 7.12, SD = 1.24, range = 6) compared to those who pledged in 2020 (mean = 7.04, SD = 1.19, range = 8). This was not statistically significant (t = 0.865, *p* = 0.367 (independent *t*-test) (Table 2).

Of the eight knowledge test questions, the statements ‘antibiotics are effective against viruses’, ‘antibiotics are effective against cold and flu’, ‘unnecessary use of antibiotics makes them become ineffective’ and ‘taking antibiotics has associated side effects such as nausea, diarrhoea and skin rashes’, were answered correctly by over 92.7% (711/767) of respondents (Supplementary Table S1). Three statements, ‘taking antibiotics may have associated risks such as drug allergy or Colitis associated with Clostridium Difficile (C.Diff)’, ‘healthy people can carry antibiotic resistant bacteria’ and ‘every person treated with antibiotics is at an increased risk of antibiotic resistant infection’ were answered correctly by more than 84.5% (648/767) of respondents. The statement ‘antibiotic resistant bacteria can spread from person to person’ was the lowest scoring question on average with 66.1% (506/767) of respondents answering correctly. Health Care Assistants (50% correct scores) and Dispensers (56% correct scores) scored lowest on this question whilst Academic Pharmacists (100% correct scores) and Pre-Registration Pharmacists (95% correct scores) scored highest. There was a statistically significant difference between job role groups and capability scores (as determined by one-way ANOVA), (F = 13.776, *p* = 0.0002).

### 3.3. Opportunity and Attitude

Opportunity and attitude questions were asked to assess whether respondents actively knew when to utilize their knowledge within their practice as well as access to local antibiotic-prescribing guidance (Supplementary Table S2). A total of 90.5% (693/767) of respondents either strongly agreed or agreed with the statement ‘I know what antibiotic resistance is’. A total of 9.2% (71/767) of respondents either disagreed or strongly disagreed with this statement. Two respondents selected either ‘undecided’ or ‘I do not know’. Only respondents who selected either ‘strongly agree’ or ‘agree’ were then directed to the set of questions regarding the opportunity to put self-reported knowledge to use (*n* = 693). The mean overall score for all respondents to these five questions was 4.16 (SD = 0.9, range = 4) equating to an average answer of ‘agree’. Of the five questions, for the statements ‘I know there is a connection between the prescribing of antibiotics and the emergence of antibiotic resistance’, ‘I know what information to give about prudent use of antibiotics and antibiotic resistance’ and ‘I have sufficient knowledge about how to use antibiotics appropriately for my current practice’, over 80% (554/693) of all respondents selected ‘strongly agree’. Over 70% (485/693) of all respondents selected either ‘strongly agree’ or ‘agree’ when answering the statement ‘I have a key role in helping control antibiotic resistance’. A total of 61.6% (423/693) of all respondents replied either ‘strongly agree’ or ‘agree’ to the statement ‘I have access to and utilise local antibiotic prescribing guidance’ (Table 3).

The four Academic Pharmacists reported ‘strongly agree’ to most statements with 75% (3/4) stating they ‘strongly agree’ with all five opportunity statements. A total of 96% (73/76) of all respondents who had been pharmacy professionals or workers for less than two years and 95% (132/139) of all respondents aged between 56 and 65 reported either ‘strongly agree’ or ‘agree’ that they knew the connection between AMR and antibiotic prescribing.

The results showed that those who pledged in 2019 had a higher overall opportunity report score (mean = 4.18, SD = 0.91, range = 4) than those who pledged in 2020 (mean = 4.15, SD = 0.82, range = 4). An independent *t*-test found that this pattern was not statistically significant, t(693) = 0.403, *p* = 0.669. Together this suggests that the year of pledge did not affect the overall opportunity statements gained by respondents.

### 3.4. Motivation

Of the 766 respondents who completed this section, 47.6% (364/766) reported that they became AGs due to mandatory workplace requirement and 43.9% (336/766) of all respondents stated that their motivation to pledge was due to professional experience with AMR. Fewer than 2% (15/766) stated that personal experience or interest led them to pledge and 6.8% (52/766) responded that the COVID-19 pandemic had highlighted the importance of antibiotic protection and led them to pledge to become AGs.

Despite the PQS changes regarding AMS not coming into place until 2020, pledging due to the mandatory requirements of workplace training was the most common answer in both the 2019 and 2020 cohorts. Forty two percent (182/429) of the 2019 pledge respondents reported that their AG training was mandatory in the workplace comparable to 54% (183/338) of 2020 pledgers.

**Figure 1:**
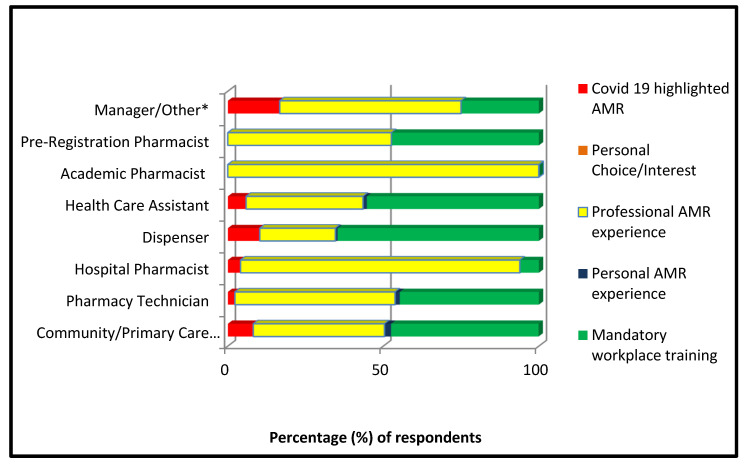
Percentage (%) of reported motivation for pledging to become an AG per job role (for full cohort). * Other was used for those who did not specify a selection/free typed job role that did not fall into a category or those that selected other e.g., CCG role.

Over 50% (68/132) of Pharmacy Technicians stated that professional experience with AMR led to their pledge and 42.2% (139/329) of Community Pharmacists also reported this motivation. A total of 64.7% (88/136) of Dispensers and 55.3% (47/85) of Health Care Assistants reported mandatory training as their main motivation for pledging (Figure 1).

For respondents whose job roles were not within community pharmacy and therefore were not incorporated in the PQS, the motivations for pledging differed significantly. As Figure 1 shows, all (4/4) Academic Pharmacists stated professional experience with AMR as their motivation to become AGs. A total of 89.8% (38/43) of Hospital Pharmacists also selected this statement. A Chi-Square test of independence was performed to examine the relationship between job role and motivation for pledging. The relationship between these variables was statistically significant. X^2^ (df 28, *n* = 766) = 87.4, *p* =< 0.00001. Community Pharmacists were more likely to select mandatory training as their motivation to pledge than those working in other areas, e.g., hospital or academia.

### 3.5. Behaviour

Respondents were firstly asked if they had recently had professional dealing with antibiotics. The statement ‘in the last week, have you dealt with prescriptions for antibiotics or given advice to patients/prescribers on antibiotic use?’ was asked, with the response either ‘yes’ or ‘no’. A total of 594 of 767 respondents (77.4%) answered ‘yes’ to this question. On answering ‘yes’, respondents were then directed to a set of recent behaviour questions (respondents answering ‘no’ were led to the next set of questions as the behaviour set would not hold appropriate relevance if they had not been in direct contact with antibiotic prescriptions or queries). As shown in Table 4, for this set of questions, Community Pharmacists, Dispensers, Pharmacy Technicians and Health Care Assistants were the over-riding cohort answering ‘yes’, resulting in them accounting for 89.5% (531/594) of the sample.

The average response for the statement ‘In the last week I have faced challenges in ad-vising patients due to COVID-19 restrictions, e.g., patients not being able to present in person’ was ‘occasionally’ (mean = 2.36 SD = 1.5, range = 5). The response most frequently selected overall for the statement ‘I am aware of local guidance on antibiotics and in the last week check prescriptions that did not comply with the prescriber before dispensing’ was ‘sometimes’ (mean = 2.8 SD = 1.8, range = 5). A total of 73.5% (435/592) respondents stated that they always ‘ensured patients had no known allergies to antibiotic medication at the time of dispensing (either verbally or by checking the Patient Medication Records). Over 40% (236/592) of all respondents also stated that they always ‘ensured health promotion materials around antibiotic use were available to patients/customers and referred to these when necessary’ and ‘in the last week if a patient requested advice on a potentially self-limiting infection, I am satisfied that I used the opportunity to provide self-care resources and gave information/advice’. The most selected response over all five behaviour statements was ‘sometimes’; however, there was a high level of standard deviation (mean = 3.36, SD = 1.07, range = 5). The results showed that those who pledged in 2019 had a higher overall knowledge score (mean = 3.39, SD = 1.06, range = 5) than those who pledged in 2020 (mean = 3.31, SD = 1.10, range = 5). Independent *t*-test t(592) = 0.937, *p* = 0.353.

### 3.6. Awareness

Respondents were asked to rank their awareness scores to key issues or initiatives around AMR on a sliding scale between one (strongly disagree) and ten (strongly agree) (Table 5). The survey participants could slide the scale across to the score they felt most reflected their answer. All 767 respondents completed the statement set and the mean response score was 7.14 (SD = 1.87, range = 9).

The mean score over all four statements for the 2019 cohort (429/767) was 7.2 (SD = 1.88, range = 9) and the mean score for the 2020 cohort (338/767) was 7.04 (SD = 1.87, range = 9). Academic Pharmacists reported the highest agreement score across all job roles with a mean score of 8.0 (SD = 1.57, range = 8). The lowest agreement mean score overall was 6.90 (SD = 2.02, range = 9) reported by Health Care Assistants. Newly registered pharmacy professionals or those who had been in the role for the shortest period scored their awareness overall as the lowest with a mean of 6.63 (SD = 2.09, range = 7). There was a strong relationship between age and self-reported awareness with those under 18 scoring an average of 6.68 on the agreement scale with this increasing with each age group to a maximum mean of 7.5 (SD = 1.24, range = 9) in those over the age of 65.

There was a high level of standard deviation for all four questions. The results showed that those who pledged in 2019 had a marginally higher awareness report score (mean = 7.23, SD = 1.88, range = 9) than those who pledged in 2020 (mean = 7.04, SD = 1.87, range = 9). An independent *t*-test found that this pattern was not statistically significant, t(765) = 1.36, *p* = 0.087. Together this suggests that the year of pledge did not affect the overall awareness statements gained by respondents.

For the question ‘Does your place of work have an antimicrobial stewardship action plan in place?’ 57.8% (443/762) of respondents answered ‘yes—and I am aware of the content’. A total of 10% (77/762) or respondents answered ‘yes—but I am not aware of the content’ and 5.6% (43/762) answered ‘no’. Of a total 762 respondents who answered this question, 25.9% (199/762) replied ‘unsure’; 60.9% (200/328) of Community Pharmacists reported they were aware of the content of their workplace stewardship plan. Managers reported a high knowledge of their workplace stewardship plans with 75% (9/12) reporting awareness.

With the question ‘Since pledging to become an AG, have you completed further training in antibiotic resistance, antibiotic use or infections?’, 62.8% (482/763) of the respondents answered ‘no’; 36.4% (119/327) of Community Pharmacists had completed further training since completion of the AG pledge, 64.6% (84/130) of Pharmacy Technicians reported they had not completed any further training since pledging and 70% (60/86) of Health Care Assistants also reported no further training on AMR since their initial pledge.

In total, 17.6% (135/767) of respondents stated that they did share their AG certificate on social media, and the most commonly reported outlet choice was Facebook. Of the respondents who answered that they did share on social media, 36–45-year-olds shared the most frequently with 35.7% (45/171) reporting that they had posted their certificate online. All of those in the under 18s and over 65s age groups reported that they had not shared their certificate on social media.

For questions regarding the PQS, 76.9% (590/766) of the total respondents said they were aware of the scheme, with 63.8% (489/586) reporting that they were aware of the addition of AMS. Notably, 181 respondents did not answer the question on the changes to the PQS, the lowest response rate of all the survey questions. A total of 75.2% (322/428) of the 2019 cohort and 79.3% (268/366) of the 2020 cohort were aware of the PQS. Of these, 85% (272/320) of the 2019 cohort and 81.6% (217/266) of the 2020 cohort were aware of the changes made to the criterion to include AMS. Dispensers accounted for the highest percentage of awareness of PQS overall across all job roles, with 86.7% (117/135) reporting knowledge of the PQS and 92% (265/288) of Community Pharmacists were aware of the addition of AMR stewardship to the criterion.

### 3.7. Exploring the Impact of Motivation on Pledging, Knowledge and Self Reported Behaviour

The Community PQS is part of the Community Pharmacy Contractual Framework and is a voluntary incentive scheme for England. Although the PQS is not mandator for pharmacy contractors (the employers/pharmacy owners), they may make the requirements mandatory for employees.

Therefore, respondents who had stipulated that they were either Academic Pharmacists or Hospital Pharmacists were removed from this analysis. The total remaining number of respondents was 714.

For an analysis of the motivational factors for pledging and their influence or impact upon subsequent self-reported behaviour and knowledge score, the motivation variables were collated into two variables from the five original selections. The two variables were ‘mandatory’ and ‘choice’. This was to explore exclusively the potential disparity between the responses from those who had pledged to be an AG as part of the PQS requirements, and those who did not. The variables ‘professional or personal experience of AMR’, ‘COVID-19 highlighted importance’ and ‘personal interest’ were collated into one variable. This variable was re-named ‘choice’. Respondents who selected ‘it was mandatory as part of my workplace training’ remained as one category, re-named ‘mandatory’. Once re-categorised, further bivariate analysis was undertaken. A total of 49.2% (351/713) of respondents pledged by ‘choice’. In total, 50.7% (362/713) of respondents pledged because it was ‘mandatory’. One respondent did not answer this question.

In 2019, before the changes to the PQS to include AMS, 46.1% (179/388) respondents pledged through mandatory requirements. In 2020, after the changes to the PQS, 56.3% (183/325) of respondents pledged through mandatory requirements. An independent *t*-test found that this was not statistically significant, t(711) = 0.722, *p* = 0.235. Together this suggests that the year of pledge did not affect the overall motivation for pledging gained by respondents.

Bivariate inferential tests were carried out to establish if the motivation for pledging to become an AG affected subsequent self-reported behaviour. Levene’s test confirmed that the assumption of homogeneity of variance was met, F(1764) = 0.91, *p* > 0.05. The group variances were not likely to be statistically significantly different. An independent *t*-test found that this pattern was not statistically significant, t(551) = −0.552, *p* = 0.291. Together this suggests that motivation for pledging did not affect subsequent mean self-reported behaviour scores. Table 3 shows individual Chi-Squared analysis for each response in the behaviour question set. The results showed that those who pledged by choice had a slightly higher average knowledge score (mean = 7.10, SD = 1.2) than those who pledged because it was mandatory (mean = 7.07, SD = 1.2). An independent *t*-test found that this difference was not statistically significant, t(764) = 0.350, *p* = 0.363, d = 0.02. Together this suggests that the motivation for the AG pledge did not affect the overall knowledge scores gained by respondents. Moreover, the Chi-Square test for independence indicated no statistically significant difference between motivation and the self-reported behaviour (Table 6).

## 4. Discussion

The current study provides unique information on the types of pharmacy workers participating in the national AG Campaign [8] with respect to the online pledge. It is the first study to analyse the relationship between an evidence-based model of behaviour change (specifically capacity, opportunity, motivation and behaviour on the whole cadre of pharmacy professionals in the UK, in relation to AMS). Furthermore, the study investigated the impact of introducing a new professional quality standard in relation to AMS through the national pharmacy quality scheme (PQS) in England, and participation and attitudes towards the AG and AMS. 

The key findings suggest that the AG Campaign provides meaningful support to pharmacy workers with respect to AMS. This was particularly so for those with professional qualifications and responsible for providing advice to patients and clinicians and ensuring the most suitable medications are dispensed. However, additional opportunities need to be developed if Pharmacy dispensers or assistants are to appreciate their role in AMS and enhance their skills and application.

Respondents had a high overall capability with regards to AMR knowledge. There was no clinically significant difference across age categories for knowledge scores. Those pledging in 2019 compared to 2020 had similar mean scores for the knowledge questions. This suggests that there was no difference in capability, as assessed by knowledge, of those responding before and after the inclusion of the AMS domain to the PQS. However, in line with previous findings, this evaluation supports the ongoing platform that pharmacy teams hold to engage with AMS [15]. It also supports the notion that the PQS advocates for the delivery of effective clinical services within pharmacy teams [11].

Previous literature states that pharmacy teams have sufficient knowledge and opportunity to communicate with prescribers to drive prudent antibiotic use [16]. This study supports this, with 72.4% (594/767) of respondents stating that ‘in the last week they had dealt with prescriptions for antibiotics or given advice to patients/prescribers on antibiotic use’. The policy statement also commented on pharmacy teams’ access to health promotional material within the workplace to further signpost patients or prescribers. Questionnaire responses reinforced this, with 67.8% (520/767) of respondents stating that their workplace had an AMS action plan in place. With broader accessibility to antibiotic prescriptions now available in the UK [17], ongoing AMS and adequate signposting is crucial.

There was a statistically significant difference in knowledge scores across job role groups. Pharmacists and Pharmacy Technicians overall scored a statistically significant higher mean score than non-registered workers (Dispensers and Health Care Assistants), who had lower knowledge scores. Previous interviews with pharmacy teams have reported that a lack of awareness of AMR and available training was the biggest barrier to stewardship [18]. This study supports this notion, with those respondents who held more qualifications reporting a higher awareness of AMR.

The use of pharmacy teams to promote the prudent use of antibiotics and support AMR efforts within the UK has been shown in previous research [6] and this project supports this notion. Pharmacy teams displayed a high overall capability towards AMS, and this has also been found in previous studies [19]. This study also supports previous commentary that the pharmacy sector has adequate capability to inform and liaise with prescribers of antibiotics and support clinical decision making [16]. The importance of gaining an understanding of pharmacy teams’ attitudes and behaviours towards AMS is highlighted. As previous studies [20] found that 23% (1985/8631) of all pledgers were from pharmacy teams, building knowledge on this cohort is vital.

Previous qualitative literature has posited that pharmacy staff overall had a low awareness of their potential contribution to AMS [15]. This study found that despite Pharmacists reporting always giving advice on prudent antibiotic use, they were largely unaware of the connection to AMS. There was also a low level of knowledge of the importance of their role in AMS and advocacy. A total of 62.8% (482/767) or respondents stated that they had not completed further training on AMR since pledging. Reflection on these findings suggests that ongoing training, rather than a one-time pledge approach, could be beneficial for pharmacy teams to encourage ongoing AMS.

To our knowledge, this project provides the first assessment of the AG campaign amongst pharmacy workers; where 52.3% (401/767) of the total respondents were Pharmacists and 47.7% (366/767) of respondents were Pharmacy Technicians, Dispensers, Health Care Assistants or Managers. It is also the first quantitative study in the UK to investigate the relationship between pharmacy professionals’ motivations for becoming an AG, their subsequent self-reported behaviour and capability and impact of the PQS.

### Strengths and Limitations

Whilst the respondents included pharmacy team members across all sectors of pharmacy, there was a low response rate overall, with only 14.6% (783/5344) of those invited to complete the study responding. However, a retrospective power calculation showed that a sample size of 170 would be enough to detect a difference in mean score of 0.5 between two groups (SD 1.15, ratio of 1.3:1 subjects in each group) with 80% power, our sample size of 783 provides 100% power to detect such a difference. Several factors could be responsible for this low uptake. The initial invitation email wording may not have been engaging or fully explanatory of the purposes of the study. This may have resulted in invitees not continuing with the questionnaire. The test–retest pilot of the questionnaire implied that the survey would take between three and seven minutes to complete. It is also possible that those that responded are those engaged and motivated to contribute to broader activities on AMS/AMR. This may have been a factor in invitees responding. The current COVID-19 pandemic has notably put pressure on pharmacy teams, and they may have had additional time constraints. Of the 783 invitees that did complete the questionnaire, 72% (442/599) of the 2019 cohort responded compared to a relatively low response from the 2020 cohort of 7.2% (341/4745). The two cohorts were emailed separately so as to yield data from each for evaluation. There was a potential error in the follow up email sent after one week. This could have led to the 2020 cohort not receiving a follow up email and could be responsible for the low response rate. Despite the questionnaire being live for an additional week to compensate for this, the 2020 response rate was low. This could suggest that the comparative samples between 2019 and 2020 are not representative and could question the validity of the motivational aspects of this project. It could also be suggested that the research time required extending to allow for further responses. As the differences in the numbers of individuals pledging to be an AG in November 2019 and November 2020 are vast, in order to reduce the likelihood of a sampling error, quota sampling or the use of a systematic random sample may have been beneficial. Furthermore, as the analyses focused on a community pharmacy, and therefore omitted hospital and academic pharmacists from some question sets, there posits a limitation to the relevance of the study on the pharmaceutical sector as a whole.

As the questionnaire source was the Antibiotic Guardian email, acquiescence bias may have influenced responses. Since the questionnaire asked respondents to report their pharmacy job role and years within the sector, it is possible that respondents would have awareness of the intentions of the questionnaire. The use of a quantitative methodology in this project however enabled a wide reach of respondents from the UK and ensured the project remained within UK COVID-19 regulations. It is also worth noting that whilst AG is UK-wide, the PQS is England only. 

In this cross-sectional study, it was not possible to confirm causality, but the results of associations are certainly positive in supporting the value of the campaign on pharmacy professionals’ capability, behaviour and opportunity to provide AMS. The study has begun to fill the gap in knowledge regarding the PQS addition of AMS and the potential effects of mandatory training to pharmacy teams. 

There is a gap in our understanding of the effect of mandatory training on subsequent behaviour and whilst this project begins to add knowledge, there is still much to explore. Future research could focus on the validity of mandatory training within community pharmacy, with a wider range of domains of the PQS explored. With incentivised and mandated training and stewardship efforts becoming increasingly utilised within healthcare, there is a necessity to continually evaluate their impact.

## 5. Conclusions

This quantitative study contributes to the body of evidence on the impact of national contractual framework including AMS and the subsequent employer requirement of mandatory training within community pharmacies in England. It also provides further evaluation of the impact of the Antibiotic Guardian campaign and its unique value in supporting AMS. There was a focus on the exploration of capability, opportunity, behaviour and awareness of pharmacy workers that had pledged to be Antibiotic Guardians in November 2019 and November 2020. Furthermore, an exploration of motivational factors for pledging was conducted, in line with changes to the Pharmacy Quality Scheme (PQS) in England to include antimicrobial stewardship activities.

There was no evidence to suggest that the catalyst for pledging affects capability. Those who pledged to become Antibiotic Guardians through personal choice reported similar behaviour to those who had pledged as a result of workplace requirement. The study was the first to our knowledge that evaluated the effects of incentivized /mandated training on community pharmacy teams. There was a statistically significant difference in the motivations for pledging across job roles. Pharmacists and Pharmacy Technicians pledged more often due to personal or professional interest; however, Dispensers and Health Care Assistants pledged mainly due to workplace mandatory requirements. This could imply that existing knowledge around AMR (through previous education when studying Pharmacy or Pharmacy Services) gives individuals a stronger sense of responsibility to advocate for prudent antibiotic use. 

This study provides a baseline for further research into the value of mandatory or incentivised training within the pharmacy sector. With the recent COVID-19 pandemic, it could be suggested that there has never been a more optimal and urgent time for an analysis of the capabilities, opportunities and motivation of pharmacy teams regarding tackling AMR. Community Pharmacy teams are playing an increasing role across the primary care pathway, including GP, dentistry and out of hours access services. There is therefore a need for ongoing research to explore how community pharmacy teams can further embed AMS activities and promote awareness of AMR as part of their clinical service. 

## Figures and Tables

**Table 1:** Age, stated gender identity, years in profession/professionally registered, pharmaceutical job role and year of pledge to AG campaign of respondents (*n* = 767).

Age (Years)	Number of Respondents	
	*n*	%
<18	4	0.5
19–25	50	6.5
26–35	157	20.5
36–45	170	22.2
46–55	187	24.4
56–65	177	2.1
>65	17	2.2
Prefer Not To Say	5	0.6
**Years In Profession/Professionally Registered**		
<2	79	10.3
3–10	275	35.9
11–20	191	24.9
21–30	96	12.5
31–40	89	11.6
41–50	16	2.1
>50	4	0.5
Prefer Not To Say	17	2.2
**Stated Gender Identity**		
Female	615	80.2
Male	144	18.8
Prefer Not To Say	8	1
**Year of Antibiotic Guardian Pledge**		
2019	429	55.9
2020	338	44.1
**Pharmaceutical Job Role**		
Community/Primary Care Pharmacist	329	42.9
Pharmacy Technician	132	17.2
Hospital Pharmacist	49	6.4
Dispenser	136	17.7
Health Care Assistant	86	11.2
Academic Pharmacist	4	0.5
Pre-Registration Pharmacist	19	2.5
Pre-Registration Pharmacy Technician	0	0
Manager/Other *	12	1.6

* Other was used for those who did not specify a selection/freetyped job role that did not fall into a category/selected other e.g., CCG role.

**Table 2:** Mean (average) scores, total respondents and Standard Deviation (SD) for Capability questions per demographic group.

Age (Years)	Total Number of Respondents		
	*n*	Mean	SD
<18	4	7	0
18–25	51	7.1	1.6
26–35	156	7.2	1
36–45	171	7.1	1.2
46–55	210	7.1	1.2
56–65	154	7	1.4
>65	17	6.7	0.9
Prefer Not To Say	4	7.5	1
**Years In Profession/Professionally Registered**			
<2	79	7.1	1.3
3–10	275	6.9	1.3
11–20	191	7.1	1.2
21–30	96	7.3	1
31–40	89	7.4	1.2
41–50	16	7.1	0.9
>50	4	7.3	0.9
Prefer Not To Say	17	7.1	0.9
**Gender Identity**			
Female	615	7.1	1.2
Male	144	7.3	1.2
Prefer Not To Say	8	7.4	1.2
**Year of Antibiotic Guardian Pledge**			
2019	429	7.12	1.24
2020	338	7.04	1.19
**Pharmaceutical Job Role**			
Community/Primary Care Pharmacist	329	7.4	0.9
Pharmacy Technician	132	7	0.9
Hospital Pharmacist	49	7.7	0.4
Dispenser	136	6.5	0.9
Health Care Assistant	86	6.6	0.8
Academic Pharmacist	4	7.7	0.5
Pre-Registration Pharmacist	19	7.5	0.7
Pre-Registration Pharmacy Technician	0	N/A	N/A
Manager/Other *	12	6.9	1.2

* Other was used for those who did not specify a selection/free typed job role that did not fall into a category/selected other e.g., CCG role.

**Table 3:** Mean (average) scores and Standard Deviation (SD) for Opportunity and Attitude questions per demographic group.

Age (Years)	Total Number of Respondents		
	*n*	Mean	SD
<18	4	3.6	0.4
18–25	49	4.3	0.8
26–35	143	4.2	0.9
36–45	158	4.3	0.7
46–55	195	4	1
56–65	138	4.3	0.9
>65	15	4.1	0.8
Prefer Not To Say	4	4.5	0.3
**Years In Profession/Professionally Registered**			
<2	78	4.2	0.7
3–10	248	4.1	0.8
11–20	178	4.2	0.9
21–30	83	4.1	0.9
31–40	79	4.5	0.5
41–50	15	4	1.2
>50	4	4.6	0.7
Prefer Not To Say	14	3.5	1.4
**Gender Identity**			
Female	560	4.1	0.9
Male	128	4.3	0.9
Prefer Not To Say	7	4.2	0.9
**Year of Antibiotic Guardian Pledge**			
2019	397	4.2	0.9
2020	258	4.2	0.8
**Pharmaceutical Job Role**			
Community/Primary Care Pharmacist	289	4.2	0.9
Pharmacy Technician	125	4	0.9
Hospital Pharmacist	43	4.7	0.4
Dispenser	128	4.1	0.9
Health Care Assistant	77	4	0.8
Academic Pharmacist	4	4.4	0.5
Pre-Registration Pharmacist	18	4.3	0.7
Pre-Registration Pharmacy Technician	0	N/A	N/A
Manager/Other *	11	4.1	1.2

* Other was used for those who did not specify a selection/free typed job role that did not fall into a category/selected other e.g., CCG role. Footnote: variables were allocated a value to analyse data (strongly agree = 5, agree = 4, undecided = 3, disagree = 2, strongly disagree = 1).

**Table 4:** Percentages (%) of statement selections for behaviour statements.

Statements Linked to Behaviour	Percentage (%) Responses						Number of Responses (sum)
	N/A	Never	Occasionally	Sometimes	Most of the Time	Always	
In the last week if a patient requested advice on a potentially self-limiting infection, I am satisfied that I used the opportunity to provide self-care resources and gave information/advice	11.3	4.9	5.1	7.1	27.2	44.3	591
In the last week I have faced challenges in advising patients due to COVID-19 restrictions	13.1	20.3	18.1	26.6	9.5	12.4	587
I am aware of local guidance on antibiotics and in the last week have checked prescriptions that did not comply with the prescriber before dispensing	15.3	17.8	12.4	10.2	15.1	29.3	590
I have ensured health promotion materials about antibiotic use were available to my patients/customers and referred to these when necessary (e.g., leaflet in nag/displayed posters)	6.4	5.4	9.5	12.7	23.6	42.4	590
In the last week when dispensing antibiotics, I have ensured the patient has no known allergies to the medication (either verbally or checking PMR)	5.1	3.2	4.2	3.5	10.5	73.5	592

**Table 5:** Average reported agreement scores of awareness statement selections from the set.

	Number of Responses	Mean Score	Average Response	Standard Deviation
I am aware of World Antibiotic Awareness Week (WAAW) which takes place each November	766	6.66	undecided	2.96
In the last year, my workplace team displayed health promotion material about antibiotic use/safety	765	8.22	strongly agree	2.37
Health promotion resources provided by agencies such as PHE are useful when providing intervention/counselling to patients/customers about antibiotic use	764	8.51	strongly agree	1.87
My workplace team has produced its own health promotion material regarding appropriate antibiotic use	766	5.19	undecided	3.19

**Table 6:** Table of individual Chi-Squared analysis for each response in the behaviour question set.

In the Last Week I have Faced Challenges in Advising Patients due to COVID-19 Restrictions			
	Value	df	Asymptotic Significance (2-sided)
Pearson Chi-Square	11.752a	5	0.038
Likelihood Ratio	11.793	5	0.038
Linear-by-Linear Association	0.763	1	0.383
N of Valid Cases	546		
a. Zero cells (0.0%) expected count of less than five. The minimum expected count was 23.63.			
**I am aware of local guidance on antibiotics and in the last week checked prescriptions that did not comply with the prescriber before dispensing**			
	Value	df	Asymptotic Significance (2-sided)
Pearson Chi-Square	10.499a	5	0.062
Likelihood Ratio	10.537	5	0.061
Linear-by-Linear Association	8.409	1	0.004
N of Valid Cases	549		
a. Zero cells (0.0%) expected count of less than five. The minimum expected count was 26.37.			
**I have ensured that health promotion materials about antibiotic use were available to my patients/customers and referred to these when necessary (e.g., leaflet in nag/displayed posters)**			
	Value	df	Asymptotic Significance (2-sided)
Pearson Chi-Square	8.340a	5	0.138
Likelihood Ratio	8.39	5	0.136
Linear-by-Linear Association	6.214	1	0.013
N of Valid Cases	549		
a. Zero cells (0.0%) expected count of less than five. The minimum expected count was 11.52.			
**In the last week when dispensing antibiotics, I have ensured the patient has no known allergies to the medication (either verbally or by checking patients records)**			
	Value	df	Asymptotic Significance (2-sided)
Pearson Chi-Square	6.181a	5	0.289
Likelihood Ratio	6.239	5	0.284
Linear-by-Linear Association	0.713	1	0.398
N of Valid Cases	551		
a. Zero cells (0.0%) expected a count of less than five. The minimum expected count was 8.76.			
**In the last week if a customer/patient requested advice on a potentially self-limiting**			
	Value	df	Asymptotic Significance (2-sided)
Pearson Chi-Square	7.049a	5	0.217
Likelihood Ratio	7.063	5	0.216
Linear-by-Linear Association	2.367	1	0.124
N of Valid Cases	550		
a. Zero cells (0.0%) expected a count of less than five. The minimum expected count was 12.52.			

## Data Availability

Not Applicable.

## Supplementary Materials

The following supporting information can be downloaded at: https://www.mdpi.com/article/10.3390/pharmacy10040098/s1. Figure S1: Ethics approval consideration was put through the University of Manchester’s ethics decision tool: https://www.training.itservices.manchester.ac.uk/uom/ERM/ethics_decision_tool/story_html5.html. Figure S2: Antibiotic Guardian (pledgers blank consent form to be contacted). Figure S3: Ques-tionnaire–consent page. Material S1: Raw output of questions included in the distributed survey and the flow. Table S1: Percentage (%) correct scores for true/false knowledge statements per pharmacy job role. Table S2: Most common (by percentage %) responses per pharmacy job role for attitude and opportunity statements.
