# Supplementary material for: Evaluating UK Pharmacy Workers’ Knowledge, Attitudes and Behaviour towards Antimicrobial Stewardship and Assessing the Impact of Training in Community Pharmacy
Source: Pharmacy (Basel). 2022 Aug 16;10(4):98. doi: 10.3390/pharmacy10040098 (PMC9414432; doi:10.3390/pharmacy10040098)
Supplement: Supplementary file 1 [file pharmacy-10-00098-s001.zip › pharmacy-1796376-supplementary.pdf]

Supplementary

Figure S1: Ethics approval consideration was put through the University of Manchester’s ethics decision tool: https://www.training.itservices.manchester.ac.uk/uom/ERM/ethics_decision_tool/story_html5.html.

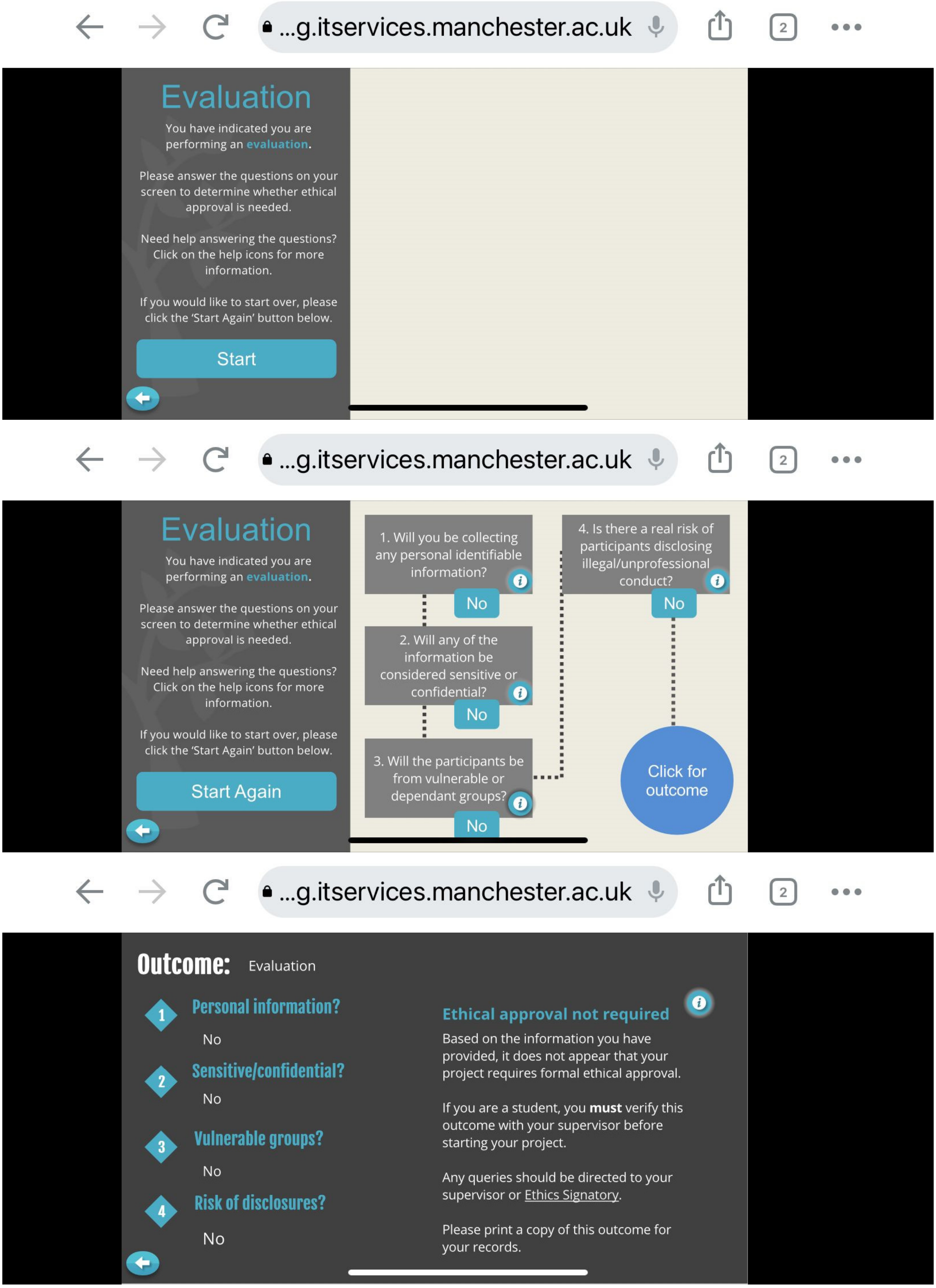

Figure S2: Antibiotic Guardian (pledgers blank consent form to be contacted).

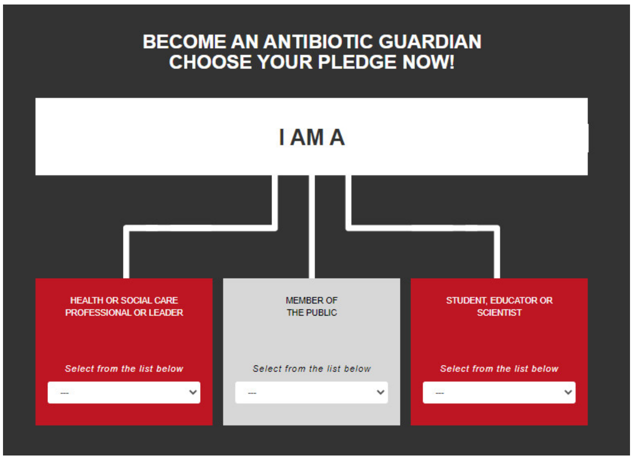

BECOME AN ANTIBIOTIC GUARDIAN
CHOOSE YOUR PLEDGE NOW!

I AM A

HEALTH OR SOCIAL CARE PROFESSIONAL OR LEADER

*Select from the list below*

MEMBER OF THE PUBLIC

*Select from the list below*

STUDENT, EDUCATOR OR SCIENTIST

*Select from the list below*

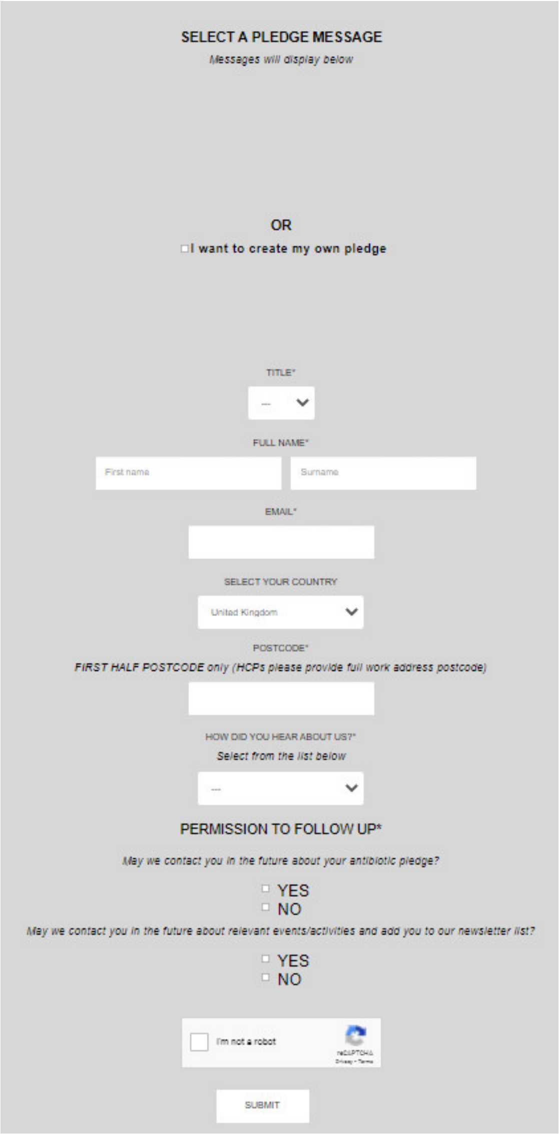

SELECT A PLEDGE MESSAGE

*Messages will display below*

OR

☐ I want to create my own pledge

TITLE*

FULL NAME*

First name

Surname

EMAIL*

SELECT YOUR COUNTRY

United Kingdom

POSTCODE*

*FIRST HALF POSTCODE only (HCPs please provide full work address postcode)*

HOW DID YOU HEAR ABOUT US?*

*Select from the list below*

PERMISSION TO FOLLOW UP*

*May we contact you in the future about your antibiotic pledge?*

☐ YES
☐ NO

*May we contact you in the future about relevant events/activities and add you to our newsletter list?*

☐ YES
☐ NO

I'm not a robot

reCAPTCHA

SUBMIT

Figure S3: Questionnaire – consent page.

BECOME AN ANTIBIOTIC GUARDIAN

Keep Antibiotics Working

## Evaluation of the Antibiotic Guardian 2019 & 2020 Campaign

[redacted] (not shared) Switch account

We are inviting members of Pharmacy Teams to complete this short survey regarding your experience with the Antibiotic Guardian campaign. Your participation is voluntary and your answers will provide us with a more detailed understanding of the campaign as part of our service evaluation.

We anticipate it will take 5 to 10 minutes to complete the questionnaire and your answers are strictly anonymous.
No identifiable information will be asked and your personal details and responses will be anonymised and stored securely.

Your responses will be analysed by Donna Seaton (MRes Public Health student) at the University of Manchester in collaboration with the Antibiotic Guardian campaign. Thank you in advance for your valuable time.

BECOME AN ANTIBIOTIC GUARDIAN

Keep Antibiotics Working

Evaluation of the Antibiotic Guardian 2019 & 2020 Campaign

[redacted] not shared) Switch account

* Required

Q1. I understand that I am being asked to participate in a questionnaire regarding my Antibiotic Guardianship pledge and that my answers may form part of an analysis by Donna Seaton on behalf of the Antibiotic Guardian campaign. I understand that all my responses are confidential and anonymous and that I can withdraw from the evaluation at any time. *

- ◯ Accept and Continue
- ◯ Decline and End Questionnaire

**Supplementary Material S1:** Raw output of questions included in the distributed survey and the flow

Evaluation of the Antibiotic Guardian Campaign 2021

Start of Block: Consent

Thank you for choosing to pledge to become an Antibiotic Guardian through www.antibioticguardian.com. There are now over 135,000 pledges made! Remember, you can always pledge again to refresh your knowledge! We are inviting members of Pharmacy Teams to complete this short survey regarding your experience with the Antibiotic Guardian campaign. Your participation is voluntary and your answers will provide us with a more detailed understanding of the campaign as part of our service evaluation. We anticipate it will take 5 to 10 minutes to complete the questionnaire and your answers are strictly anonymous. No identifiable information will be asked and your personal details and responses will be anonymised and stored securely. Your responses will be analysed by Donna Seaton (MRes Public Health student) at the University of Manchester in collaboration with the Antibiotic Guardian campaign. Thank you in advance for your valuable time.

Q1. I understand that I am being asked to participate in a questionnaire regarding my Antibiotic Guardianship pledge and that my answers may form part of an analysis by Donna Seaton on behalf of the Antibiotic Guardian campaign. I understand that all my responses are confidential and anonymous and that I can withdraw from the evaluation at any time.

o Accept and continue

o Decline and end questionnaire

Skip To: End of Survey If Q1 = Decline and end questionnaire

End of Block: Consent

Start of Block: Demographics

Q2 When pledging, you selected you were a member of a Pharmacy Team: Select your pharmacy group: (select all that apply)

▢ Primary care pharmacist

▢ Hospital pharmacist

▢ Community pharmacist

▢ Academic pharmacist

▢ Pharmacy technician

▢ Pharmacy assistant

▢ Pharmacy dispenser

▢ Pharmacy counter assistant

▢ Pre-registration pharmacist

▢ Pre-registration pharmacy technician

Q3 what was your year of registration? (if not registered, what year did you begin working in the pharmacy sector?)

o Prior to 1970

o 1970 - 1979

o 1980 - 1989

o 1990 - 1999

o 2000 - 2009

o 2010 - 2019

o After 2019

o I don't know/Prefer not to say

Q4 what is your year of birth?

Q5 Select your gender identity

o Male o Female o Other o Prefer not to say

End of Block: Demographics

Start of Block: Knowledge

Q6. To what extent do you agree/disagree with the following statement?

Strongly disagree (1)

Disagree (2)

Undecided (3)

Agree (4)

Strongly agree (5)

I do not understand/not applicable (6)
I know what antibiotic resistance is
Skip To: Q8 If Q6 = I know what antibiotic resistance is [strongly disagree]
Q7. To what extent do you agree/disagree with the following statements?
Strongly disagree (1)
Disagree (2)
Undecided (3)
Agree (4)
Strongly agree (5)
I do not understand/not applicable (6)
I know there is a connection between the prescribing of antibiotics and the emergence of antibiotic resistance
I know what information to give to individuals about prudent use of antibiotics and antibiotic resistance
I have sufficient knowledge about how to use antibiotics appropriately for my current practice
I have a key role in helping control antibiotic resistance
I have access to and utilise local antibiotic prescribing guidance
Q8. Please answer whether you believe these statements are true or false
True (1) False (2)
Antibiotics are effective against viruses
Antibiotics are effective against cold and flu
Unnecessary use of antibiotics makes them become ineffective
Taking antibiotics has associated side effects such as nausea, diarrhoea and skin rashes
Taking antibiotics may have associated risks such as drug allergy or Colitis associated with Clostridium Difficile (C.Diff)
Every person treated with antibiotics is at an increased risk of antibiotic resistant infection
Antibiotic resistant bacteria can spread from person to person
Healthy people can carry antibiotic resistant bacteria
End of Block: Knowledge
Start of Block: Behaviour
Q9. In the last week, have you dealt with prescriptions for antibiotics or given advice to patients/prescribers on antibiotic use?
o Yes o No
Skip To: Q11 If Q9 = No
Q10. To what extent have you acted within the following statements?
never (1) occasionally (2) sometimes (3) most of the time (4) always (5) N/A (6)
In the last week if a customer/patient requested advice on a potentially self-limiting infection, I am satisfied that I have used this opportunity to provide self-care resources and give information/advice
I am aware of local guidance on antibiotics and in the last week checked prescriptions that did not comply with the prescriber before dispensing
In the last week I have faced challenges in advising patients due to Covid 19 restrictions (e.g. patient not being able to present in person)
I have ensured that health promotion materials about antibiotic use were available to my patients/customers and referred to these when necessary (e.g. leaflet in bag/displayed posters)
In the last week when dispensing antibiotics, I have ensured the patient has no known allergies to the medication (either verbally or by checking patients records)
End of Block: Behaviour
Start of Block: Capability
Q11 Where 0=strongly disagree and 10=strongly agree - to what extent do you score the following questions?
I am aware of World Antimicrobial Awareness Week (WAAW) which takes place every November
In the last year, my workplace team displayed health promotion material about antibiotic use/safety
Health promotion resources provided by agencies such as Public Health England are useful when providing intervention/counselling to patients/customers about antibiotic use
My workplace team has produced its own health promotion material regarding appropriate antibiotic use
Page Break
Q12 Does your place of work have an antimicrobial stewardship action plan in place?
o Yes, I am aware of the content
o Yes, but I am not aware of the content
o No
o I am not sure
Q13 Since pledging to become an Antibiotic Guardian, I have completed further training in antibiotic resistance, antibiotic use or infections

o Yes o No o I am not sure
Q14 Did you share your Antibiotic Guardian certificate on social media?
o Yes, on Facebook (1)
o Yes, on Twitter (2)
o Yes, on LinkedIn (3)
o Yes, on more than one social media channel (4)
o No (5)
End of Block: Capability
Start of Block: Motivation
Q15 Which of the following was the MAIN reason you chose to pledge to become an Antibiotic Guardian? (select one)
o I have personal experience with antibiotic resistance
o I have professional experience with antibiotic resistance
o Personal choice through general interest in stewardship or for professional development
o It was mandatory for me (as part of my work place training/expectations)
o The Covid 19 pandemic drew attention to the importance of antibiotic resistance and I wanted to play my part in supporting the campaign
Q16 Are you aware of the Pharmacy Quality Scheme?
o Yes (1) o No (2)
Skip To: End of Survey If Q16 = No
Page Break
Q17 Did you know that an Antimicrobial Stewardship criterion has been added to the Pharmacy Quality Scheme?
o Yes (1) o No (2)
End of Block: Motivation
Debrief:
Many thanks for completing the questionnaire, service evaluation provides valuable feedback to help continually improve and support the Antibiotic Guardian campaign. If you would like more information on the evaluation or the results of the survey, please email donna.seaton@postgrad.manchester.ac.uk or the project supervisor roger.harrison@manchester.ac.uk. Your feedback is highly valuable. Thank you.

Supplementary Table S1: Percentage (%) correct scores for true/false knowledge statements per pharmacy job role.

| Knowledge Statement | Correct Answer | Community/ Primary Care Pharmacist | Pharmacy Technician | Hospital Pharmacist | Dispenser | Health Care Assistant | Academic Pharmacist | Pre-Registration Pharmacist | Manager/ Other* |
|---|---|---|---|---|---|---|---|---|---|
| **Antibiotics are effective against viruses** | FALSE | 97 | 97 | 100 | 83 | 87 | 100 | 84 | 83 |
| **Antibiotics are effective against cold and flu** | FALSE | 97 | 97 | 100 | 83 | 87 | 100 | 95 | 100 |
| **Antibiotic resistant bacteria can spread from person to person** | TRUE | 77 | 56 | 90 | 50 | 49 | 100 | 95 | 58 |
| **Healthy people can carry antibiotic resistant bacteria** | TRUE | 91 | 92 | 98 | 84 | 82 | 100 | 100 | 83 |
| **Taking antibiotics has associated side effects such as nausea, diarrhoea and skin rashes** | TRUE | 99 | 95 | 98 | 98 | 96 | 100 | 95 | 100 |
| **Every person treated with antibiotics is at an increased risk of antibiotic resistant infection** | TRUE | 85 | 84 | 92 | 79 | 86 | 100 | 84 | 83 |
| **Taking antibiotics may have associated risks such as drug allergy or Colitis associated with Clostridium Difficile (C.Diff)** | TRUE | 93 | 83 | 100 | 67 | 79 | 100 | 100 | 83 |

Supplementary Table S2: Most common (by percentage %) responses per pharmacy job role for attitude and opportunity statements.

Table 5: Most common (by percentage %) responses per pharmacy job role for opportunity statements

| Knowledge Statement | Primary Care/ Community Pharmacist | Pharmacy Technician | Hospital Pharmacist | Dispenser | Health Care Assistant | Academic Pharmacist | Pre-Registration Pharmacist | Manager/ Other* |
|---|---|---|---|---|---|---|---|---|
| I know there is a connection between the prescribing of antibiotics and the emergence of antibiotic resistance | 69.9% strongly agree | 58.9% strongly agree | 88.4% strongly agree | 49.2% strongly agree | 55.8% strongly agree | 100% strongly agree | 83.3% strongly agree | 54.5% strongly agree |
| I know what information to give about prudent use of antibiotics and antibiotic resistance | 58.1% strongly agree | 38.7% strongly agree | 62.8% strongly agree | 46.5% agree | 54.5% agree | 75% strongly agree | 50% agree | 54.5% agree |
| I have sufficient knowledge about how to Use antibiotics appropriately for my current practice | 55.7% strongly agree | 46.4% agree | 48.8% strongly agree | 47.2% agree | 46.6% strongly agree | 100% strongly agree | 55.6% agree | 54.55% strongly agree |
| I have a key role in helping control antibiotic resistance | 49.5% strongly agree | 48.8% strongly agree | 73.8% strongly agree | 42.4% agree | 39% agree | 50% strongly agree | 55.6% strongly agree | 45.5% agree |
| I have access to and utilise local antibiotic prescribing guidance | 35.8% agree | 33.1% agree | 79.1% strongly agree | 42.5% agree | 26.3% agree | 50% strongly agree | 33.3% strongly agree | 60% agree |
